# Use of Diluted Pyroligneous Acid as a Gargle

**Published:** 1852-05

**Authors:** John Evans

**Affiliations:** Professor of Obstetrics, &c., in Rush Medical College


*Use of Diluted Pyroligneous Acid as a Gargle.* By John Evans, M. D., Professor of Obstetrics, &c., in Rush Medical College.—I have for several years been using diluted Pyroligneous Acid as a gargle in cases of inflammation of the fauces and tonsils with better success than any other article that I have prescribed.

I put a teaspoonful of the acid obtained from thes hops into a wine-glass of water and direct the patient to gargle the throat frequently with it.

In the sore throat caused by exposure, so common throughout the country, it generally relieves the soreness and stiffness felt in swallowing very promptly.

In chronic inflammation, with or without ulceration, of the throat, I have found it a very valuable remedy.

In the sore throat of Scarlatina it has generally afforded a very prompt amelioration of this symptom of the disease.

In several cases of habitual tonsilitis, by using this gargle freely at the commencement of the disease, I have been able to arrest the progress of the iuflammation and secure a resolution.

Its use is not unpleasant; it is safe, even if used for hours continuously, and has an additional advantage in removing the fœtor of the breath.—*North Western Medical and Surgical Journal.*

